# Depending on intensity, exercise improved or worsened pathology in a model of prodromal Parkinson’s disease

**DOI:** 10.1038/s41531-025-01200-y

**Published:** 2025-11-23

**Authors:** Leonie Baldauf, Malte Feja, Milos Stanojlovic, Till Strowig, Christian Visscher, Eva Schaeffer, Daniela Berg, Julia Hankel, Franziska Richter

**Affiliations:** 1 Department of Pharmacology, Toxicology, and Pharmacy; University of Veterinary Medicine Hannover, Hannover, Germany; 2 Center for Systems Neuroscience Hannover (ZSN), Hannover, Germany; 3 Department of Neurobiology, Institute for Biological Research Siniša Stanković - National Institute of Republic of Serbia, University of Belgrade, Belgrade, Serbia; 4 Department of Microbial Immune Regulation, Helmholtz Center for Infection Research, Braunschweig, Germany; 5 Centre for Individualised Infection Medicine (CiiM), a joint venture between the Helmholtz-Centre for Infection Research (HZI) and the Hannover Medical School (MHH), Hannover, Germany; 6 Institute for Animal Nutrition, University of Veterinary Medicine Hannover, Hannover, Germany; 7 Department of Neurology, University Hospital Schleswig-Holstein, Christian-Albrechts University, Kiel, Germany

**Keywords:** Parkinson's disease, Parkinson's disease

## Abstract

Exercise is increasingly applied as non-pharmacological intervention in Parkinson’s disease (PD). While evidence suggests beneficial effects, the mechanisms, optimal intensity, and potential risks remain unclear. This study investigated the effects of treadmill exercise on motor and non-motor symptoms, alpha-synuclein pathology, neuroinflammation, and gut microbiota composition in Thy1-aSyn transgenic mice, a model of prodromal PD. Moderate (5 m/min) or intensive (12.5–15 m/min) treadmill exercise three times a week for one month delayed motor deficits, with improvements in beam-walking performance and gait coordination. Intensive training attenuated anxiety-like behavior and reduced phosphorylated alpha-synuclein accumulation in the substantia nigra, but not in limbic regions, demonstrating region-specific pathology modulation. Despite absent baseline neuroinflammation, intensive exercise increased microglial reactivity in the amygdala and hippocampus. Additionally, exercise increased fecal microbiota diversity across genotypes, with selective enrichment of Lachnospiraceae. These findings suggest that exercise modulates PD-related pathology and motor symptoms, although intensity-dependent limbic microgliosis warrants further investigation.

## Introduction

Parkinson’s disease (PD) is the second most common progressive neurodegenerative disease affecting 2–3% of people over the age of 65 years. The classical hallmarks of PD are appearance of Lewy bodies and neurites in the brain of patients, and loss of dopaminergic neurons in the substantia nigra pars compacta (SNc) leading to the signature motor symptoms such as resting tremor, bradykinesia, and rigidity^1,2^. In recent years, it has been recognized that PD is a multilayered disease that affects many different brain regions, neuronal populations and signaling pathways, including mitochondrial dysfunction, autophagy imbalance, neuroinflammation and protein misfolding^3–7^. While the cause of PD remains unknown, alpha-synuclein is thought to play an important role in disease pathogenesis and progression^7^. Alpha-synuclein is present in Lewy bodies and its accumulation causes extensive pathology that correlates with neuronal dysfunction^8–10^.

There is currently no pharmacological treatment available to halt or reverse neurodegenerative processes in PD, but there are multiple clinical trials ongoing for symptomatic and disease-modifying therapeutics^11^. Alpha-synuclein-related pathology remains a key therapeutic target^12,13^. Unfortunately, at the time when PD is diagnosed based on the cardinal motor symptoms, the majority of dopamine neurons are already lost and underlying disease processes are far advanced^14^. Therefore, effective disease-modifying intervention has to start at an early, prodromal or even preclinical phase of the disease^15^. This, however, requires the identification of persons at risk of developing the disease, which has complex ethical implications for an incurable disease. It has been seen that changes in lifestyle, such as physical exercise, may ameliorate disease progression in clinical PD and thus could also be offered to people at risk or in the prodromal phase of the disease^16–19^.

While it is undisputed that physical activation improves clinical motor scores, muscle strength, balance, gait, posture, cognition, general well-being and quality of life measures, information regarding how and to what extent exercise affects PD onset is limited^16,20–23^. A meta-analysis of studies quantifying the effect of different dosages of physical exercise on PD revealed that moderate to vigorous physical activity decreases the risk of PD among men intensity-dependently, while women benefited less. According to these calculations, a person must walk at least 1 h/day at a speed of 5 km/h to decrease the risk to develop PD by 50%^24^. Imaging data supported that aerobic exercise stimulates functional and structural neuroplasticity in both motor and cognitive brain networks in PD. However, while global brain atrophy was reduced, structural integrity of the substantia nigra remained unchanged^25^. Among the potential mechanisms, an increase in neurotrophic factors is thought to be a major driver of exercise-induced neuroprotection^26^. However, the precise mechanisms remain unknown. If high-intensity exercise is required for efficacy, then it remains important to exclude deleterious effects, such as stress-induced increase in neuroinflammation^27–30^. Toxin-induced animal models of PD were used to identify the effects of exercise on striatal dopamine signaling or post-synaptic spine density^26,31,32^. Still, studies on disease pathogenesis in the prodromal phase, including potential deleterious effects, are scarce.

Here, we determined the effects of moderate versus intensive exercise on the progression of behavioral deficits and pathology in the Thy1-aSyn mouse model of synucleinopathy (Line 61), which is well-characterized for therapeutic studies^33^. Mice were assessed at 1–3 months of age, which resembles the prodromal stage of PD, with alpha-synuclein pathology and fine motor deficits already present in transgenic mice, but prior to overt loss of dopamine and hypokinesia^33^.

## Results

### Exercise slowed down progression of motor and non-motor phenotype

**Fig. 1:**
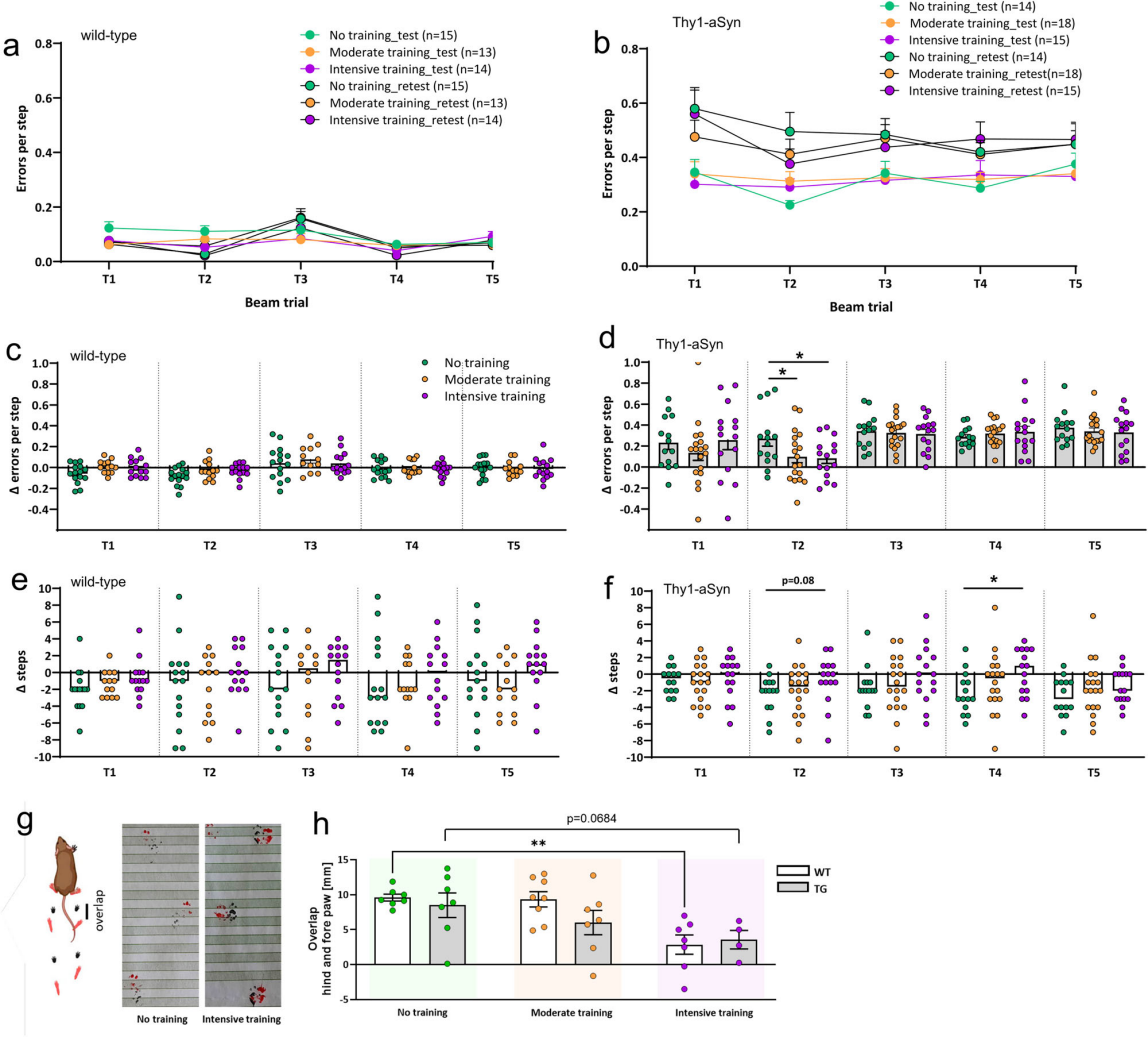
Exercise delays progression of motor phenotype in Thy1-aSyn transgenic mice. The effects of different exercise regimens (no, moderate or intensive training) on motor phenotypes of wild-type (WT) control and transgenic (TG) mice are depicted. **a**, **b** Beam performance assessed as errors per step, calculated for each trial, before (test) and after (retest) the exercise phase in WT and TG, respectively. Change in error rate (**c**, WT; **d**, TG) and step count (**e**, WT; **f**, TG) between test and retest. **g**, **h** Gait analysis based on the overlap of hind and fore paws, illustrated by footprint examples from untrained and intensively trained TG mice. Data are shown as mean +/−SEM; two-way ANOVA with Sidak’s multiple comparisons for post-hoc analysis; *P < 0.05, **P < 0.01 moderate/intensive vs. no training (**d**, **f**, **h**; exercise effect); *P* between 0.05 and 0.1 indicates a trend.

Exercise on a treadmill over 4 weeks was well-tolerated in Thy1-aSyn transgenics and wild-type littermates at both, moderate (5 m/min) and intensive (12.5 m/min–15 m/min interval) training regimens. There were no effects on body weight (Fig. S1a, b) and no increase in morbidity compared to the no-training group, in which mice were sitting in the turned-off treadmill during the training session. Both genotypes were able to learn running on the treadmill during the adaptation phase of two weeks. At 1–3 months of age, as assessed here, Thy1-aSyn mice are at a prodromal stage of progression, with alpha-synuclein pathology already accumulating, but prior to overt loss of dopamine. As described previously, challenging tests were used to detect the early and progressive fine motor deficits^33^, recorded as errors per step on the challenging beam test prior to exercise (‘test’) and 4 days after the last training session (‘re-test’) over 5 repeated trials (Fig. 1). Progression of this motor deficit between 1.5 and 3 months of age was evident for transgenic mice (on average 0.3 error/step increase between test and re-test), while wild-types made few errors independent of age or exercise regimen (Fig. 1a, b). To determine effects of exercise on progression, delta of errors per step between re-test and test were calculated and analyzed (Fig. 1c, d). Exercise had no effect on errors per step in wild-type mice (Fig. 1c). In Thy1-aSyn transgenics, moderate and intensive exercise reduced progression of this motor deficit compared to the no-training group in the second out of 5 trials (Fig. 1d, *p* < 0.05). As observed previously, the first out of 5 trials showed the highest variability in transgenic mice, as they need to remember how to perform the test (Fig. 1d trial 1). Exercise did not improve the performance in the first out of 5 trials, probably indicating that there was also no positive impact on the cognitive ability of transgenic mice to remember the task. Reduction of errors in transgenics under moderate or intensive exercise in trial 2 is related to motor improvement. Interestingly, mice could not sustain this improved performance in the following trials 3–5, suggesting that it requires effort and muscle strength to avoid errors. If exercise would have improved muscle strength in this model, mice could have potentially sustained this increase in coordination over the five repetitions. There were no overt effects on general fitness and muscle strength recorded with the pole test, where mice need to grip the pole to turn and climb down (data not shown). To analyze whether trained mice differ in their stepping pattern on the challenging beam, the delta in numbers of steps between re-test and test was calculated. In general, mice increased in weight and body size between 1.5 and 3 months of age which increased step length and thereby reduced numbers of steps (negative delta in Fig. 1e, f). In wild-type mice, this led to variable alterations of numbers of steps between test and retest without significant effects of exercise (Fig. 1e). As observed previously, transgenic mice made less steps on the beam with phenotype progression compared to wild-type^34^, with lower inter-individual variability, as they did not adjust their steps to the grid overlay. Interestingly, intensive exercise reduced this phenotype towards more steps to perform the test, indicative of a difference in stepping pattern (Fig. 1f, *p* < 0.05). This is further corroborated by footprint analysis to quantify the overlap of the fore- and hind-paws. Under extensive exercise, mice of both genotypes changed their stepping pattern towards zero overlap, which signifies that the hind-paw steps at the position of the respective fore-paw (Fig. 1g, h). This improved motor coordination in both genotypes coincided with reduced progression of the fine motor deficits of transgenics on trial 2 of the beam test.

**Fig. 2:**
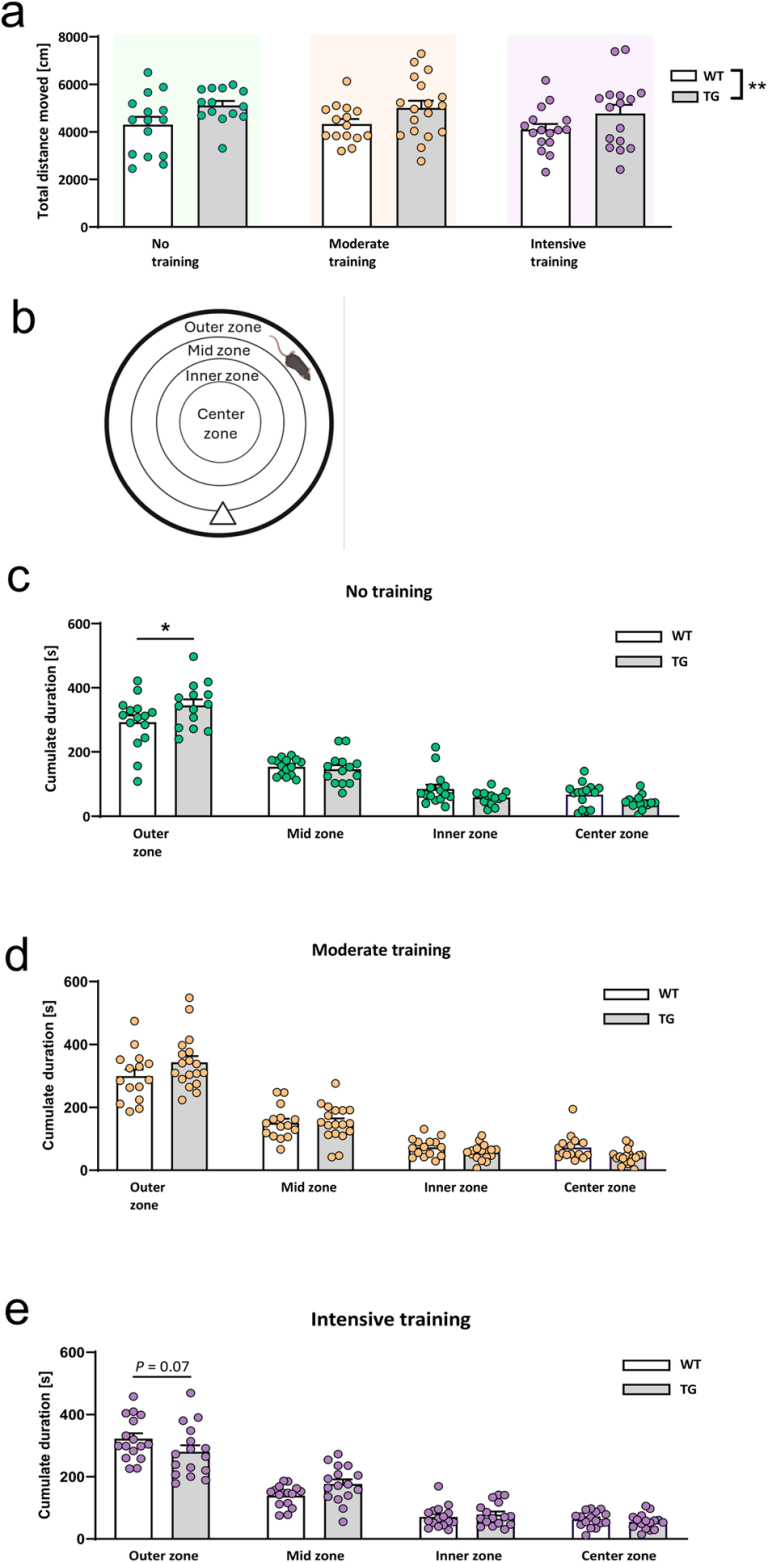
Exercise effects on the anxiety phenotype in Thy1-aSyn transgenic mice. The effects of different exercise regimens (no, moderate or intensive training) on anxiety phenotype of wild-type (WT) control and transgenic (TG) mice are depicted in the open field. **a** Overall locomotor activity measured as total distance moved is unchanged. **b**–**e** Activity levels assessed in untrained (**c**), moderately trained (**d**), and intensively trained (**e**) mice across different open field zones (**b**). Data are shown as mean +/−SEM; two-way ANOVA with Sidak’s multiple comparisons for post-hoc analysis; *P < 0.05 TG vs. WT (**c**; genotype effect); **P < 0.01 TG vs. WT (**a**; main effect of genotype); *P* between 0.05 and 0.1 indicates a trend.

The open field test was performed following exercise training at 3 months of age to record effects on general activity and anxiety (Fig. 2). There were no effects of exercise on general activity, whereby transgenic mice generally exhibited higher activity (Fig. 2a). This is in line with previous observations, as Thy1-aSyn transgenic mice are hyperactive at 6 months of age, when extracellular dopamine levels are increased, which progresses to PD-like deficits with hypolocomotion at 14 months of age, when dopamine loss is evident^33,35^. As expected, transgenic Thy1-aSyn mice show increased anxiety-like phenotype in the open field, expressed as higher duration in the outer zone (Fig. 2b, c, thigmotaxis). This phenotype was absent in the moderately or intensively trained groups (Fig. 2d, e), with the intensively trained transgenic mice now tending to spend less time in the outer zone compared to wild-type (Fig. 2e, *p* = 0.07).

### Exercise reduced alpha-synuclein pathology in the substantia nigra

**Fig. 3:**
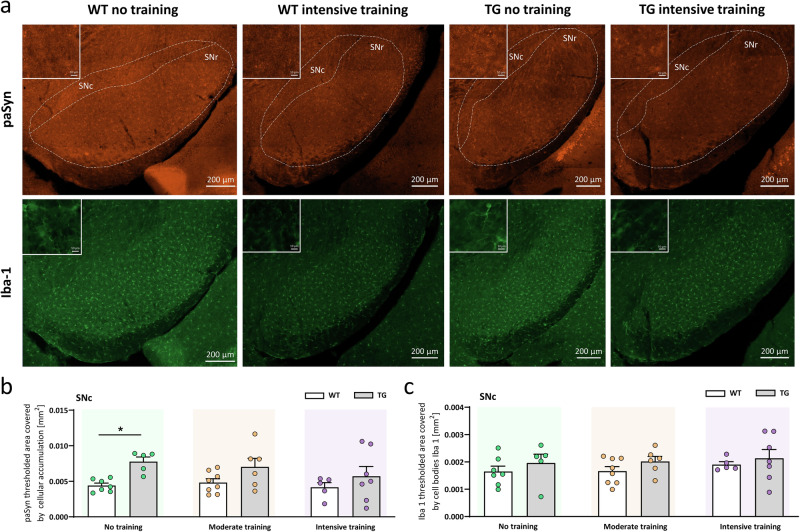
Exercise reduces alpha-synuclein pathology in the substantia nigra of Thy1-aSyn mice. **a** Immunofluorescent staining of Ser129 phosphorylated human alpha-synuclein (paSyn) and Iba-1-positive microglia in the substantia nigra pars compacta (SNc) of wild-type (WT) control and transgenic (TG) mice that received no or intensive training; 200x total magnification, scale bars are as indicated (200 µm, insets: 10 µm). Quantification of **b** paSyn (fluorescent area thresholded to intracellular accumulation) and **c** Iba-1-positive microglia (fluorescent area thresholded to cell bodies) in SNc of WT and TG mice that received no, moderate, or intensive training; data are shown as mean + SEM; two-way ANOVA with Sidak’s multiple comparisons for post-hoc analysis; *P < 0.05, TG vs. WT (genotype effect).

In Thy1-aSyn transgenic mice, similar to observations in PD patient brains, phosphorylated alpha-synuclein increases in the substantia nigra pars compacta^33^, where dopaminergic neurons degenerate predominantly. This was already evident at 3 months of age for the no-training control group, with a clear discrimination in levels of phosphorylated alpha-synuclein in transgenic mice versus wild-type littermates (Fig. 3a, b, no training, p < 0.05). Under moderate exercise, phosphorylated alpha-synuclein levels remained increased on average, but the variability in the trained group increased (Fig. 3b, moderate training, n.s.). In intensively trained mice, the majority of transgenic mice showed phosphorylated alpha-synuclein at wild-type level (Fig. 3b, intensive training). Impact on neuroinflammation was recorded by staining for microglia, the resident immune cells of the brain. In previous studies, Thy1-aSyn transgenic mice showed increased microglia reactivity in the substantia nigra at about 6 months of age^12,36^. Here, at 3 months of age, this pathology was not yet fully developed (Fig. 3c, no training). Neither moderate nor intensive exercise significantly influenced the progression of neuroinflammation in transgenic mice (Fig. 3c).

### Exercise increased microgliosis in limbic brain regions of Thy1-aSyn mice

**Fig. 4:**
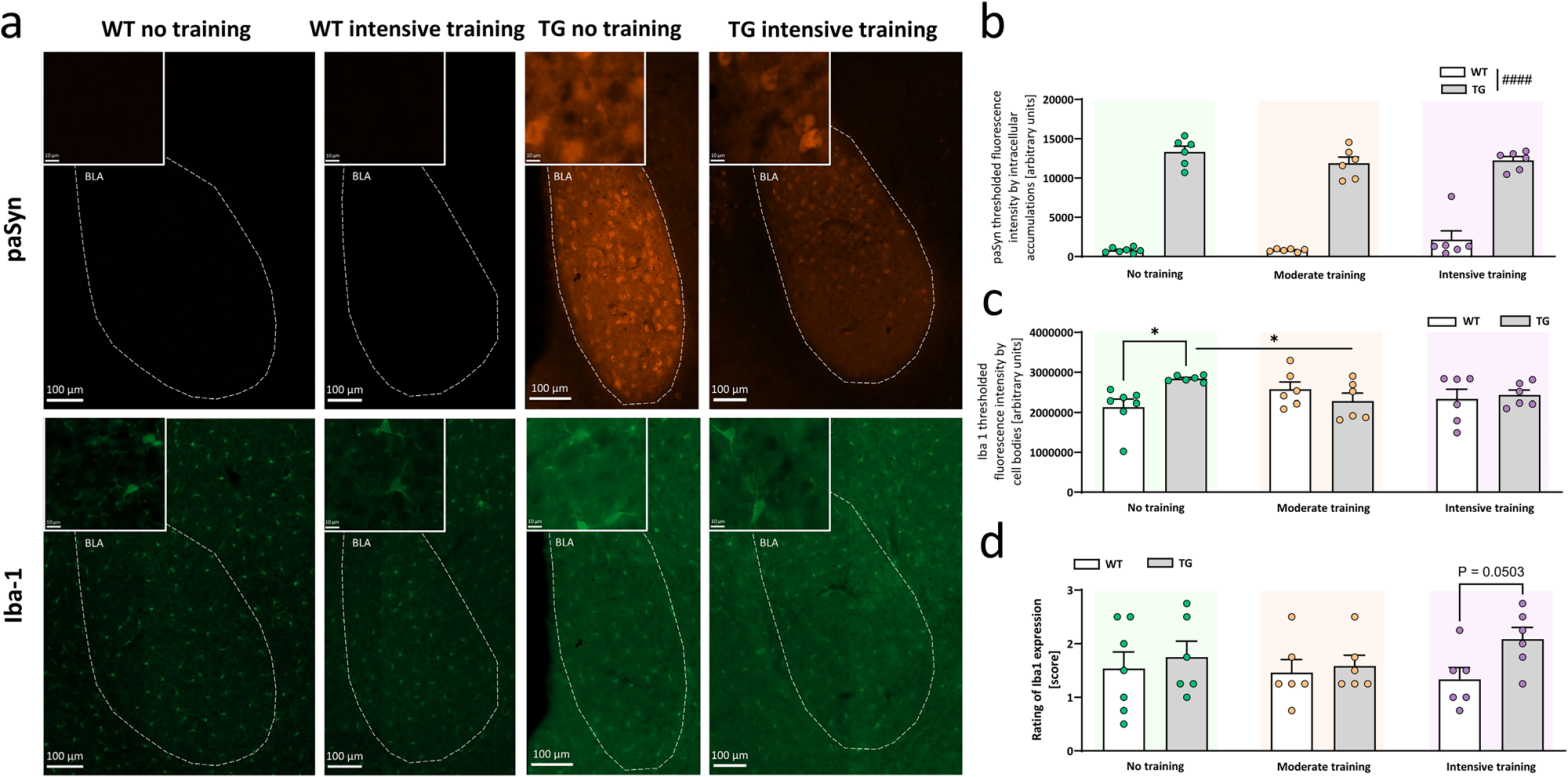
Intensive exercise tends to increase microgliosis in the basolateral amygdala of Thy1-aSyn mice. **a** Immunofluorescent staining of Ser129 phosphorylated human alpha-synuclein (paSyn) and Iba-1-positive microglia in the basolateral amygdala (BLA) of wild-type (WT) control and transgenic (TG) mice that received no or intensive training; 200x total magnification, scale bars are as indicated (100 µm, insets: 10 µm); fixed display range across all images (0–8000 digital gray value). Quantification of **b** paSyn (fluorescence intensity thresholded to intracellular accumulation) and **c** Iba-1-positive microglia (fluorescence intensity thresholded to cell bodies), and **d** semiquantitative rating of Iba1 expression in BLA of WT and TG mice that received no, moderate or intensive training; data are shown as mean + SEM; two-way ANOVA with Sidak’s multiple comparisons for post-hoc analysis; ^####^*P* < 0.0001 TG vs. WT (main effect of genotype); *P < 0.05, TG vs. WT (genotype effect), no vs. moderate (exercise effect); *P* between 0.05 and 0.1 indicates a trend.

**Fig. 5:**
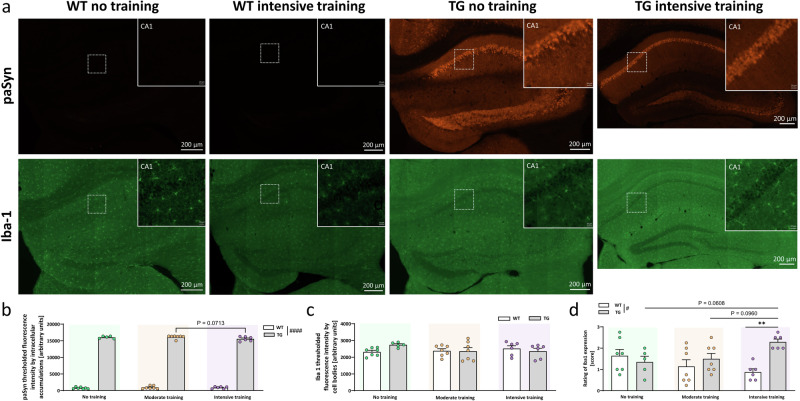
Intensive exercise increases microgliosis in the hippocampal CA1 region of Thy1-aSyn mice. **a** Immunofluorescent staining of Ser129 phosphorylated human alpha-synuclein (paSyn) and Iba-1-positive microglia in the hippocampal cornu ammonis (CA)1 region of wild-type (WT) control and transgenic (TG) mice that received no or intensive training; Region of interest (CA1, indicated by dashed rectangle, 200x total magnification, scale bars are as indicated (200 µm, insets: 20 µm); fixed display range across all images (0-5000 digital gray value). Quantification of **b** paSyn (fluorescence intensity thresholded to intracellular accumulation) and **c** Iba-1-positive microglia (fluorescence intensity thresholded to cell bodies), and **d** semiquantitative rating of Iba1 expression in CA1 of WT and TG mice that received no, moderate or intensive training; data are shown as mean + SEM; two-way ANOVA with Sidak’s multiple comparisons for post-hoc analysis; ^#^*P* < 0.05, ^####^*P* < 0.0001 TG vs. WT (main effect of genotype); *P* between 0.05 and 0.1 indicates a trend.

Non-motor deficits in PD are at least in part caused by pathology in limbic brain regions such as the basolateral amygdala (BLA) or the hippocampus. Thy1-aSyn mice develop alpha-synuclein pathology and microgliosis in the BLA and the CA1 region of the hippocampus, and increased anxiety and fear^12,37^. As expected, untrained transgenic mice showed increased phosphorylated alpha-synuclein in the BLA (Fig. 4a, b; Fig. S2) and CA1 (Fig. 5a, b; Fig. S3). Exercise did not overtly impact this pathology. Microgliosis in the BLA was already evident at the young age of 3 months in untrained transgenic Thy1-aSyn mice compared to wildtypes (Fig. 4a, c). Of note, this increase in Iba1-staining intensity was not observed under moderate or intensive exercise, with moderately trained transgenic mice showing less Iba1-staining intensity compared to untrained transgenics (Fig. 4c). However, rating of reactivity phenotype (larger cell bodies, prominent processes) by a blinded observer indicated a strong trend to increased microgliosis in intensively trained transgenic mice (Fig. 4c, *p* = 0.0503). This indicates an exercise dose dependent effect on Iba1 expression and morphology of microglia in the BLA of Thy1-aSyn mice. A similar impact of exercise on microgliosis was also observed in the CA1 region of the hippocampus (Fig. 5). Thy1-aSyn mice did not yet show significantly increased microgliosis in absence of training (Fig. 5a, c), which is in line with previous observations where this brain region is affected only at later stages of progression in PD. However, there were trends to increased rated microglia reactivity in transgenic mice under intensive exercise versus moderate and no training groups (Fig. 5d, *p* = 0.0608 vs. moderate training, *p* = 0.0960 vs. no training). Together with a non-significant numerical reduction in microgliosis in trained versus untrained wildtypes, this resulted in a significantly elevated microglia reactivity state in transgenics versus wildtype mice under intensive training (Fig. 5d).

### Exercise increased fecal microbiota diversity independent of genotype

**Fig. 6:**
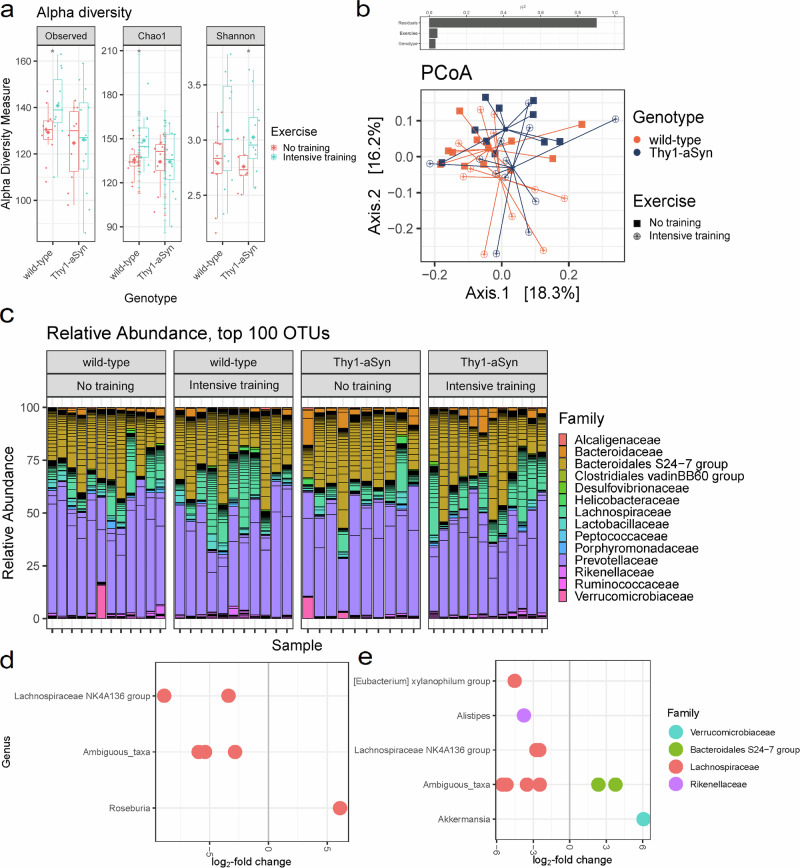
Intensive exercise increases fecal microbiota diversity independent of genotype. **a** Box-plots showing Observed Species, Chao1 and Shannon indices in fecal samples of mice depending on exercise intensity, separately for the genotype (wild-type, Thy1-aSyn). **b** Permutational multivariate analysis of variance (PERMANOVA) on Bray–Curtis distances was used to quantify the contribution of the factors Genotype and Exercise to the differences in microbial composition of the samples (above). Bray–Curtis dissimilarity-based principal coordinate analysis (PCoA) was performed on fecal pellets of mice. Different colors represent samples obtained from different genotypes (wild-type, Thy1-aSyn). Different point shapes represent samples of a varying training intensity. Lines connect samples obtained from the same group (below). **c** Bar charts represent the relative abundances of the dominant bacterial families in fecal samples of mice. DESeq2 analysis of differentially abundant operational taxonomic units (OTUs, significance threshold for padj alpha < 0.05) in fecal samples comparing **d** wild-type and **e** Thy1-aSyn mice that received no training vs. intensive training. Each dot represents a single OTU identified within a genus and grouped by color according to which taxonomic family the OTU originates. A positive log_2_-fold change indicates enrichment under no training compared to intensive training. A negative log_2_-fold change indicates enrichment under intensive training compared to no training.

Under intensive exercise for one month, bacterial richness and diversity increased in fecal samples of mice (Table 1, Fig. 6a).

**Table 1:** Alpha diversity (means ± SD) in fecal samples from mice of different genotypes and exercise intensities using the species richness estimators Observed Species, Chao1 and Shannon index

	wild-type		*p*-value	Thy1-aSyn		*p*-value
Exercise	no training *n* = 12	intensive training *n* = 11		no training *n* = 10	Intensive training *n* = 12	
Observed	129 ± 10.7	141 ± 15.1	0.047 *	125 ± 16.2	126 ± 22.9	0.867
Chao1	136 ± 11.1	149 ± 17.3	0.039 *	134 ± 17.3	134 ± 22.0	0.997
Shannon	2.79 ± 0.32	3.09 ± 0.49	0.097	2.76 ± 0.18	3.03 ± 0.34	0.040 *

*p-value < 0.05.

### Significant effect of training on gut microbial community

Only the factor Exercise has a significant effect on the bacterial composition of the samples (Table 2). Testing the prediction that microbiota would cluster by Exercise yielded a significant *p*-value, however, the *R*^2^ value was low (0.04318), suggesting poor separation of the communities by this variable alone, as also visualized in Fig. 6b.

**Table 2:** Permutational multivariate analysis of variance (PERMANOVA) results analyzing differences in microbiota composition in fecal samples from mice due to the factors Genotype and Exercise based on Bray-Curtis dissimilarities

	DF	SumsOfSqs	F.Model	*R*^2^	Pr(>F)
Genotype	1	0.1097	1.4952	0.03287	0.127
Exercise	1	0.1441	1.9644	0.04318	0.021*
Genotype:Exercise	1	0.0755	1.0293	0.02263	0.403
Residuals	41	3.0077		0.90132	
Total	44	3.3370		1.00000	

*p-value < 0.05.

### Identification of specific taxa associated with the training intensity

Fecal microbiota was dominated at the phylum level by Bacteroidetes (80.1% ±11.4) and Firmicutes (18.2% ±11.1). Relative abundances of bacterial phyla in every sample are shown in Fig. 6c.

The number and the differentially abundant features in the pairwise comparisons of the exercise level and genotype are listed in Tables 3 and 4 and shown in Fig. 6d, e.

**Table 3:** Number of differentially abundant features identified in the pairwise comparisons of the groups

Obtained samples	Comparison	*p* < 0.05	pFDR < 0.05 & abs(log2FC) > 1
wild-type	no vs intensive training	28	6
Thy1-aSyn	no vs intensive training	29	11
no training	wild-type vs Thy1-aSyn	33	9
intensive training	wild-type vs Thy1-aSyn	18	6

Raw *p*-values were adjusted using the method of Benjamini and Hochberg^91^ to control a false discovery rate (FDR) of 5%. Additionally, a cutoff for the log_2_-fold change of ±1 was set.

**Table 4:** Differentially abundant OTUs in the pairwise comparisons of the groups

OTU	log2FoldChange	pvalue	padj	Phylum	Family	Genus
**wild-type, no training vs intensive training**						
OTU_43	−8.86	7.55e−08	1.32e−05	Firmicutes	Lachnospiraceae	Lachnospiraceae NK4A136 group
OTU_53	−5.39	1.10e−06	9.62e−05	Firmicutes	Lachnospiraceae	Ambiguous_taxa
OTU_87	−2.83	6.38e−05	0.00372	Firmicutes	Lachnospiraceae	Ambiguous_taxa
OTU_131	−3.39	0.00041	0.01784	Firmicutes	Lachnospiraceae	Lachnospiraceae NK4A136 group
OTU_109	6.095	0.00083	0.02612	Firmicutes	Lachnospiraceae	Roseburia
OTU_264	−5.93	0.00090	0.02612	Firmicutes	Lachnospiraceae	Ambiguous_taxa
**Thy1-aSyn, no training vs intensive training**						
OTU_53	−5.52	2.91e−08	4.04e−06	Firmicutes	Lachnospiraceae	Ambiguous_taxa
OTU_19	6.072	2.33e−05	0.00161	Verruco-microbia	Verruco-microbiaceae	Akkermansia
OTU_90	−4.52	3.47e−05	0.00161	Firmicutes	Lachnospiraceae	[Eubacterium] xylanophilum group
OTU_234	2.36	9.75e−05	0.00300	Bacteroidetes	Bacteroidales S24-7 group	Ambiguous_taxa
OTU_36	3.76	0.00011	0.00300	Bacteroidetes	Bacteroidales S24-7 group	Ambiguous_taxa
OTU_144	−2.50	0.00039	0.00877	Firmicutes	Lachnospiraceae	Lachnospiraceae NK4A136 group
OTU_157	−3.51	0.00044	0.00877	Firmicutes	Lachnospiraceae	Ambiguous_taxa
OTU_264	−5.20	0.00100	0.01679	Firmicutes	Lachnospiraceae	Ambiguous_taxa
OTU_29	−2.74	0.00144	0.02220	Firmicutes	Lachnospiraceae	Lachnospiraceae NK4A136 group
OTU_66	−2.46	0.00323	0.04487	Firmicutes	Lachnospiraceae	Ambiguous_taxa
OTU_61	−3.78	0.00390	0.04908	Bacteroidetes	Rikenellaceae	Alistipes
**no training, wild-type vs Thy1-aSyn**						
OTU_142	−6.67	1.73e−06	0.00029	Firmicutes	Lachnospiraceae	Ambiguous_taxa
OTU_19	6.11	3.36e−06	0.00029	Verruco-microbia	Verruco-microbiaceae	Akkermansia
OTU_38	2.67	0.00023	0.01264	Bacteroidetes	Bacteroidales S24-7 group	Ambiguous_taxa
OTU_118	5.21	0.00029	0.01264	Tenericutes	Anaero-plasmataceae	Anaeroplasma
OTU_26	2.19	0.00093	0.03207	Proteobacteria	Helico-bacteraceae	Helicobacter
OTU_126	3.30	0.00115	0.03207	Bacteroidetes	Porphyromo-nadaceae	Parabacteroides
OTU_109	−5.85	0.00130	0.03207	Firmicutes	Lachnospiraceae	Roseburia
OTU_70	−4.53	0.00199	0.04300	Firmicutes	Lachnospiraceae	Ambiguous_taxa
OTU_5	1.89	0.00225	0.04320	Bacteroidetes	Bacteroidaceae	Bacteroides
**intensive training, wild-type vs Thy1-aSyn**						
OTU_234	−2.72	6.14e−05	0.01061	Bacteroidetes	Bacteroidales S24-7 group	Ambiguous_taxa
OTU_25	3.08	0.00021	0.01767	Bacteroidetes	Bacteroidaceae	Bacteroides
OTU_58	−4.73	0.00031	0.01767	Bacteroidetes	Bacteroidales S24-7 group	Ambiguous_taxa
OTU_36	−3.26	0.00045	0.01932	Bacteroidetes	Bacteroidales S24-7 group	Ambiguous_taxa
OTU_226	−4.35	0.00155	0.04638	Firmicutes	Lachnospiraceae	Ambiguous_taxa
OTU_131	−2.36	0.00161	0.04638	Firmicutes	Lachnospiraceae	Lachnospiraceae NK4A136 group

*Raw *p*-values were adjusted using the method of Benjamini and Hochberg to control a false discovery rate (FDR) of 5%.

OTU_53 and OTU_264 were repeatedly identified in as significantly different between training intensity in both genotypes. OTU_53 and OTU_264 were enriched in samples of mice that were trained intensively (Table 4). Both OTUs represents bacterial species that are assigned to the family *Lachnospiraceae* within the phylum Firmicutes. Generally, OTUs belonging to the family *Lachnospiraceae* are mainly enriched within the fecal microbiota of mice due to training.

## Discussion

This study presents evidence for disease-modifying capacity of exercise in an established model of PD. One month training intensity-dependently and moderately delayed progression of motor deficits, while alpha-synuclein pathology was reduced in the SNc only. However, in the limbic system microglia appeared more reactive under high intensity exercise. This is contrary to the expectation that intensive exercise is beneficial in PD, as microgliosis is generally considered an important driver of disease pathology. Importantly, exercise altered the microbiota composition, which may have contributed to the observed behavioral effects.

In Thy1-aSyn mice, human alpha-synuclein expression initiates in the first weeks after birth, with related pathology subsequently occurring in various brain regions as the mice age. The Thy-1 promoter drives transgene expression in neurons^38^, but resulting pathology still impacts certain brain regions more pronounced, including the SNc. Alpha-synuclein aggregation is accumulating in SNc and limbic brain regions over the first months of age, followed by overt microgliosis at about 6 months of age^33,39^. Dopamine homeostasis is progressively altered, with a peak of increased extracellular dopamine levels and hyperactivity at 6 months, developing into a decline in striatal dopamine levels, significant at 14 months of age. At the late stage of progression, Thy1-aSyn mice present with PD-like symptoms such as hypokinesia and catalepsy^35^. However, similar to PD patients at early disease stages, young transgenic mice show motor deficits if presented with challenging tasks, such as the challenging beam test, starting in the first months of age^34^. While these deficits are present prior to overt dopamine loss, they are likely related to observed alterations at the corticostriatal synapse^33,35,39^. Importantly, this loss of fine motor control is very different from the overt loss of motor function observed in models with spinal cord pathology, as discussed previously^33,35,39^. In this study, mice were between 1.5 and 3 months old, whereby exercise training was applied after a two weeks adaptation period continuously over one month, three times per week, between 2 and 3 months of age. For the choice of a moderate versus intensive treadmill regimens, previous studies in mice were consulted^30,40–46^, considering an aerobic versus anaerobic regimen. Aerobic training is a longer, less intense form of exercise that challenges endurance, such as walking^23,47–49^. In anaerobic training, energy is provided through anaerobic glycolysis, and it involves more intense, shorter activities that require maximum strength and performance, such as interval training^23,50,51^. Aerobic training is ideal for improving endurance and the cardiovascular system, while anaerobic training is more focused on strength, speed, and muscle development^50,52^. In the present study, the moderate training (final speed of 5 m/min, 0% incline) leans towards an aerobic regimen to mimic fast walking in humans, whereby the intensive interval training (12.5 m/min with an increase to 15 m/min, 0% incline) replicates challenging anaerobic regimens, both of which are applied in PD patients^23,47^. The control group consisted of animals placed only on the stationary treadmill, thus impact of novel environment and handling on outcome data can be excluded. Motor deficits in Thy1-aSyn mice prompted us to not further increase the maximum speed and incline of the treadmill, at has been done in other studies^46^. Mice readily performed the task after the 14 day adaptation period without any obvious signs of distress, and with the expected body weight gain, appearing to find the treadmill enriching. Importantly, the electric shock grid was not applied, mice learned to run without negative reinforcement during the adaptation period.

The challenging motor tests to record progression of the motor phenotype in Thy1-aSyn mice were performed before and after the exercise regimen. As expected, the well-established deficit in errors per step was observed, which worsened over the course of one month in untrained transgenic mice. While exercise was not able to reduce the errors per step in transgenics compared to controls, it delayed the progression of this deficit, visible in a significant lower delta between re-test and test compared to untrained transgenics. This was the case for both regimens, moderate and intensive, and is in line with observations in patients, such as the subtle but detectable effects of high-intensity treadmill training on motor symptom progression in de novo PD^23^. In depth analysis via footprints supported that the stepping of mice changed under exercise, which likely improved their fine motor coordination and their ability to step onto the grid and avoid foot slips. Interestingly, in the open field test, intensively trained transgenic mice tended to spend less time at the walls than wildtypes, which is a measure of anxiety significantly increased in untrained transgenics^12,37^. Thus, the absence of this previously documented phenotype points to disease-modifying effects in the limbic brain regions, as observed previously with pharmacological strategies^12,33^.

Reduction of phosphorylated alpha-synuclein, a marker of pathology^53,54^, in the SNc corroborates a disease-slowing effect of exercise on pathology in brain circuits relevant for motor function. However, no effects on alpha-synuclein pathology were observed in the limbic system, i.e. the amygdala and the hippocampus, suggesting that this effect is brain region specific. A potential explanation could be brain region specific increase of neurotrophic factors, which could be determined in future studies.

As expected, at 3 months of age, Thy1-aSyn mice did not yet show overt microgliosis in SNc and the limbic system compared to control wild-type^36^. In the SNc, microglia reactivity was not altered by exercise. Conversely, in the amygdala and hippocampus of intensively trained Thy1-aSyn mice, these brain resident immune cells showed a more reactive phenotype with larger cell body and fewer, more prominent processes, compared to controls. Of note, in the BLA moderate exercise appeared to reduce Iba-1 staining intensity of microglia cells, which might indicate a reduction of neuroinflammation. Together these data show, that exercise may alter microglia reactivity intensity-dependently in Thy1-aSyn mice, but only in the limbic system. It is well-established that neuroinflammation increases in response to stress, such as forced treadmill exercise, lifestyle changes or systemic inflammation^27–30,55^. In limbic brain regions microglia may be especially sensitive to stress induced by high intensity exercise. However, it is unclear whether the observed reactive microglia could be detrimental to the surrounding neuronal tissue, or are part of an exercise-initiated protective response to alpha-synuclein pathology. However, there are multiple studies discussing the role of microgliosis in PD, overall supporting a positive correlation of microglia reactivity and alpha-synuclein pathology^28,56–60^. In 6 months old Thy1-aSyn mice, increased fear correlates with microgliosis and reduction in parvalbumin interneurons^37^. Regardless, exercise did not enhance alpha-synuclein pathology despite increase in microglia reactivity in the limbic system in the present study. Furthermore, Thy1-aSyn mice appear less anxious in the open field test under intensive exercise. Thus, microglia reactivity apparently does not increase this non-motor symptom. Conversely, in previous studies, mice in which forced exercise induced brain damage were more anxious and showed clear signs of distress^30^. Apart from these considerations, it is difficult to predict whether mice would respond with less or more fear to alterations of the amygdala-hippocampal circuitry, as fear learning could be impaired as well^58^. Given the limited understanding of underlying mechanisms and long-term impacts, specific care should be taken that exercise in PD patients remains at a level where patients feel comfortable and not chronically stressed or forced. Further studies are urgently warranted to characterize limbic microgliosis under intensive exercise in Thy1-aSyn mice, and the short and long-term impacts on disease progression.

Several mechanisms have been proposed for the putative neuroprotective effects of physical activity: for instance, physical activity in animal models of PD has been shown to increase the production of various growth factors and receptors, maintain dopaminergic function, and reduce inflammation and oxidative stress^61,62^. In toxin-induced rodent models of PD, treadmill activity preserved striatal dopamine, whereby forced disuse of limbs increased pathology^63,64^. Interestingly, and different from observations in the present study, suppression of microglia reactivity by treadmill exercise was identified as potential mechanism in MPTP toxin-induced mice^46^. Among the potential mechanisms of disease-modifying capacity of exercise, gut microbiota should be considered. The gut microbiome is required for Thy1-aSyn mice to develop motor deficits, microglia activation, and aSyn pathology, and alpha-synuclein pathology can transmit from gut to brain in mouse models^65,66^. Remarkably, colonization of Thy1-aSyn mice with microbiota from PD patients increases motor impairments and neuroinflammation compared to microbiota transplants from healthy humans^66^. Meta-analysis of the PD gut microbiome revealed depletion of bacteria belonging to the *Lachnospiraceae* family, important short-chain fatty acids (e.g., butyrate) producers, as the most consistent alteration to healthy individuals^67^. Decreased levels of *Lachnospiraceae*-produced butyrate are related to epigenetic changes in neurons from PD patients and to the severity of their depressive symptoms^68^. A recent report highlighted a potential role for the intestinal microbiome in influencing exercise performance. Members of the *Lachnospiraceae* family emerged as drivers of enhanced exercise performance in mice^69^. Intriguingly, bacteria belonging to *Lachnospiraceae* were mainly enriched within the fecal microbiota of our intensively trained Thy1-aSyn mice, indicating that exercise-induced alterations in gut microbiota might beneficially affect PD-related brain pathology.

Our study is performed in an animal model, which has inherent limitations regarding translation to patients. Research using quadruple mice performing a repetitive forced running paradigm over one month cannot fully translate to the diversity of exercise regimens in PD patients performed over years. For instance, activities include tango and folk dancing^70–72^, strength and endurance training^73,74^, Tai Chi^75^, balance training^76,77^, power training/high-speed yoga^78^, as well as aqua exercise^79^. For discussion of our data, we referred to clinical studies with treadmill exercise^23^, which appear most comparable. The presented data provides first insights into the disease-modifying potential of such straightforward repetitive regimens, and how the dose and potentially stress-levels may impact the outcome. Of note, Thy1-aSyn mice carry the transgene on the X-chromosome, thus only male mice were used in this study and sex differences could not be determined.

In conclusion, the present data in a model of alpha-synuclein overexpression supports a disease-modifying capacity of exercise. In PD patients, physical activation improves motor- and non-motor symptoms as well as quality of life measures in the short-term, and showed the potential to slow down disease progression in the long term^16,20–23^. The present study fully supports these observations and suggests changes in microbiota composition and a reduction in alpha-synuclein pathology in the SNc as potential mechanisms. However, the presented data also cautions that high intensity exercise has the potential to increase microglia reactivity in the limbic system in presence of alpha-synuclein pathology, with yet unknown impact on disease progression. This requires more studies in the future and suggests that a broad range of symptoms should be assessed in clinical studies with different exercise regimens, and patients should be closely monitored over extended periods of time.

## Methods

### Animals and study design

**Fig. 7:**
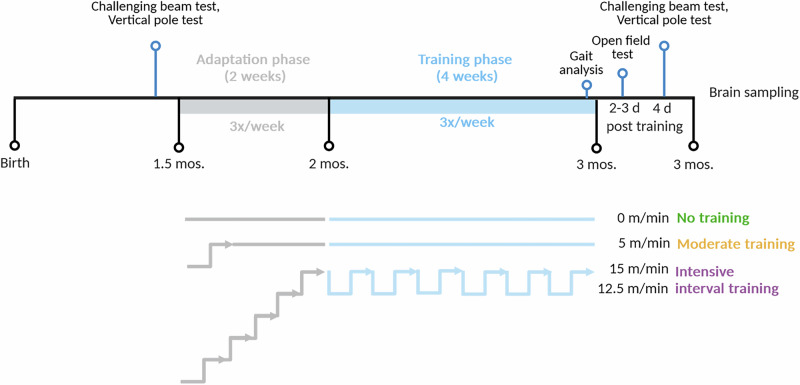
Study design overview. Mice underwent motor behavior testing (challenging beam, vertical pole test) at 6 weeks of age, followed by a 2-week adaptation phase and a 4-week exercise phase (3 sessions/week, 15 min/session). During adaptation, mice were trained to run on a motorized treadmill and gradually acclimated to the speed used in the exercise phase. Mice were assigned to one of three exercise groups: sedentary (stationary treadmill), moderate training (continuous running at 5 m/min), or intensive training (interval training: 10 min at 12.5 m/min, 5 min at 15 m/min). Motor and anxiety behavior (challenging beam, vertical pole, and open field test) were assessed 2–4 days after the last exercise session. At 3 months of age, 2 days after the final behavioral tests, mice were euthanized for brain histology analysis.

Male Thy1-αSyn mice (line 61, on a hybrid C57BL/6-DBA/2 background) and non-transgenic wild-type littermates (WT) were used. Mice had unrestricted access to standard lab chow and water and were housed within a conventional open cage system with woodchip bedding and nesting material. The light/dark cycle was reversed, with a dark (nocturnal) phase from 11:00 to 23:00 and a light (diurnal) phase from 23:00 to 11:00. All animal experiments were conducted in accordance with the guidelines of the German Animal Welfare Act and approved by the local authorities (license number: 20/3517). The experimental unit was a single animal (ARRIVE guidelines). Genotyping of mice was performed before weaning and re-genotyping to guarantee correct group allocations were performed at the end of the experiment. A total of 96 mice were used in the study. Sample sizes were determined based on a priori power analysis^39^ (*n* = 13–18/group; details see figures). Mice were systematically allocated to experimental groups based on genotype. There were no significant differences in body weight or other baseline characteristics between the experimental groups. At 1–3 months of age, as assessed in this study, Thy1-aSyn transgenic mice are at a prodromal stage, with alpha-synuclein pathology and non-motor as well as fine-motor deficits developing progressively, but prior to overt loss of dopamine^33,35,39^. The study design is depicted in Fig. 7. At 6 weeks of age mice were tested for fine motor skills (test), followed by a 2 week adaptation period to learn running on the treadmill, followed by 4 weeks of exercise 3x/week for sessions of 15 min. Two to 4 days after the last exercise, mice were tested for motor and anxiety behavior. Two days after the last behavioral tests, at 3 months of age, mice were euthanized to process brains for histology.

### Exercise regimen

Mice were trained for running on a 5-lane motorized treadmill (Model 60A SM 400-LS-Z, maze engineers, USA). The industrial made treadmill needed to be optimized by removing pointed and sharp corners of the plastic material, and by rearranging the metal grid at the end of each lane such that there remained no space between the belt and the grid, to avoid tail injuries. Mice were encouraged to keep running by gently nudging their backs with a wooden pen, but allowed pausing for up to three seconds on the metal grids. After each run, the lane separators and the treadmill were cleaned with 70% ethanol. Corresponding control groups were placed on the switched-off treadmill, serving as sedentary controls to match the handling and environmental exposure experienced by the exercising groups and to ensure that any observed effects can be attributed to exercise rather than differences in handling or context.

Acceleration was set to 100 m/min² and incline to 0% for all experimental groups. Animals were systematically assigned to three different exercise regimens as follows: (a) sedentary group sitting in the turned-off treadmill, (b) moderate training group running constantly at 5 m/min and (c) intensive training group performing interval training with 10-minute running at 12.5 m/min followed by 5 min at 15 m/min. Speeds and regimens were chose to model moderate versus intensive training based on literature values^30,40–46^.

The beginning of the training regime in each group represents an adaptation phase of 2 weeks in which the animals can get used to the treadmill and to the running speed. The exercise phase over one month followed consecutively. In both phases, animals were trained three times a week for 15 minutes each. Adaptation was done by splitting the 15 min training time and gradually introducing the animals to the different speeds until they reach the final regimen assigned to each group as stated above (Fig. 7).

### Behavioral testing and collection of fecal samples

Behavioral testing was done at time-points indicated in Fig. 7, with challenging beam and pole test performed before exercise at 5–6 weeks of age and after exercise at 12 weeks of age to measure effects on progression of motor deficits in Thy1-aSyn mice, and open field test performed only once after exercise at 12 weeks of age to compare general activity and anxiety phenotype between experimental groups. Protocols were performed as described previously^12,34,80,81^. Both tests were conducted in the dark phase of the light cycle, under dim light, following prior habituation to the room for one hour. All behavioral equipment was cleaned using 70% alcohol, wiped down and allowed drying between animals. For the challenging beam test, each mouse was trained to traverse a narrowing, square, horizontal beam for two days and tested on the third day with a meshed grid placed on the beam for 5 trials. Errors per step constituted foot slips on the meshed grid, which were analyzed together with the time to cross the beam manually at slow motion video recordings. For the vertical pole test, each mouse was trained for two days to turn and climb down the vertical pole without falling and tested on the third day for 5 trials by recording the time to turn and finish the test. The second test round was done without prior training as mice remember the tests. For footprint analysis, mouse paws were colored with ink and the mouse placed on top of absorbent paper to run through a half tube, as described previously^82^. The overlap of fore- to hindpaw footprint was measured and recorded. The open field apparatus consisted of a white plastic cylinder (55 cm in diameter; 60 cm in height) and a tripod-mounted camera was used to record behavior from above. The mouse was placed in the cylinder and its behavior was recorded over one 10-min trial. Automated analysis was performed using the three-point discrimination tool by EthoVision XT 9 software (Noldus) to track the mice for distance moved, velocity, and residence in periphery or center which was defined as inner circle of 15 cm diameter. Mice released fecal pellets during this 10-min exploration period, which were collected and stored at −80 °C for microbiota analysis.

### Histology

The day following the last behavioral test mice were euthanized with an overdose of pentobarbital and transcardially perfused with 4% paraformaldehyde for immunohistochemistry. Brains were removed, post-fixed in 4% paraformaldehyde overnight, placed in sucrose gradient (10%, 20% and 30%), washed, frozen on dry ice and stored at −80 °C. Free floating sections were processed for immunohistochemistry according to previously published standard protocols^12,37^. Brains were cut into coronal sections (40 μm) on a cryostat and sections collected. Sections were washed with 0.05 M tris-buffered saline (TBS) for 10 min three times. After thorough washing in TBS (3×10 min) sections were further processed with the standard protocol below. After washing, sections were blocked using 10% normal goat serum (NGS) in TBS. Sections were then incubated overnight in 2% NGS/0.5% TritonX in TBS with a primary antibodies (1:500, Table 5) for 24 h at 4 °C. Sections were washed with 0.05 M TBS three times for 10 min each wash, and then incubated in Alexa Fluor-conjugated secondary antibodies (1:500, Table 5) in 5% NGS for two hours at room temperature. After another round of washing, sections were mounted on glass slides and covered with ProLong®Gold anti fading medium with DAPI (Cell Signaling, Danvers, MA). ZEISS Axio Observer microscope and Zen Zeiss software (Carl Zeiss, Oberkochen, Germany) was used to capture low (10×) to high (20 or 40x) magnification images. Maximum intensity projections of five 5 µm Z-stack images using 20x magnifications were used for the analysis. Images were analyzed for area covered by immune-reactivity by outlining the area of interest using ImageJ and Fiji software as described previously^12,37^. To minimize variation in fluorescence staining and imaging, sections of experimental groups to be statistically compared were all run in the same immunohistochemical staining with previously established specific primary antibodies followed by imaging procedure under constant exposure parameters. Thus, variation in fluorescence intensity reflects mainly protein expression, therefore fluorescent background was not subtracted. Alpha-synuclein is broadly expressed, not allowing to define a stain-free brain region with certainty to be used for background fluorescence intensity measurements. For quantification of specific staining inside an outlined region, fluorescence thresholds were manually set in Fiji. For total region-of-interest fluorescence, the entire dynamic range of pixel intensities was included for measurement. For specific signals (i.e., paSyn accumulations and Iba1-positive somata), the upper threshold was fixed at the maximal intensity, while the lower threshold was gradually increased according to the histogram distribution until only the relevant signal was retained, thereby reducing subjective bias. Then the integrated density was calculated by Fiji, which is the measured fluorescence intensity times the stained (threshold) area divided by the total region of interest. Area covered by specific staining (in mm^2^) or arbitrary fluorescence units are the reported outcome data for alpha-synuclein staining. For comparative imaging, identical display ranges were applied across all experimental groups to ensure unbiased visualization. Consequently, regions with inherently lower staining intensity (e.g., WT samples in BLA and CA1) appeared with little or no visible signal, consistent with the quantitative analysis. To aid anatomical orientation without affecting quantitative interpretation, supplementary figures (Figs. S2, S3) show WT images with adjusted scaling of digital gray values (20-fold reduction in the basolateral amygdala, 10-fold reduction in the hippocampus relative to TG images), provided solely for tissue outline visualization and not for between-group intensity comparison. Additional WT insets with rescaled regions of interest were included for visibility. Morphology of Iba1-positive microglia as an indication of their reactivity to alpha-synuclein overexpression was assessed semi-quantitatively following a rating scale with score 0 being all microglia with a small cell body and several processes, and score 1–3 describing the percentage of microglia with large cell bodies and few processes as follows: score 1 for about up to 20% of microglia, score 2 for about up to 50% of microglia and score 3 for more than 50% microglia with this morphology, as described previously^12,37^.

**Table 5:** Antibodies used

Vendor	Antigen	Species / direct label	Order-No
**Primary antibodies**			
Sigma	parvalbumin	mouse	P3088
Synaptic Systems	parvalbumin	guinea pig	195004
Abcam	pS129-aSyn	rabbit	51253
Novus Biologicals	TH	rabbit	NB300-109
Synaptic Systems	Iba1	guinea pig	234 004
Synaptic Systems	Iba1	guinea pig	234 308
Synaptic Systems	PSD95	rabbit	N3783
Synaptic Systems	synaptophysin	mouse	101111
BD Transduction Labs	alpha-synuclein	mouse	610787
**Secondary antibodies**			
Invitrogen	AF 555	goat anti mouse	A32727
Jackson Laboratory	AF 488	goat anti mouse	115-545-146
Jackson Laboratory	AF 488	goat anti rabbit	111-545-144
Invitrogen	AF 488	goat anti guinea pig	A11073
Invitrogen	AF 647	goat anti rabbit	A21244
Invitrogen	AF 647	goat anti guinea pig	A21450
Invitrogen	AF 647	goat anti rabbit	A32733

### DNA extraction, 16S rRNA gene sequencing and analysis

DNA was isolated from fecal samples using the ZymoBIOMICS 96 MagBead DNA Kit (Zymo Research Europe GmbH, Freiburg, Germany). The hypervariable V4 region of the 16S rRNA gene was amplified following established protocols^83^ utilizing the primer F515/R806. The amplicons were sequenced on the Illumina MiSeq platform (PE300). The Usearch8.1 software package facilitated the merging, quality control, and clustering of the reads. The command “fastq_mergepairs” with the parameter “fastq_maxdiffs 30” was used to merge the reads. Chimeric sequences were identified and removed using the “cluster_otus” command (-otu_radius_pct 3) and the Uchime command within the Usearch8.1 workflow. Quality filtering was applied using “fastq_filter” (-fastq_maxee 1) with a minimum read length of 200 bp. Reads were clustered into operational taxonomic units (OTUs) with 97% identity. OTU clusters and representative sequences were determined using the UPARSE algorithm^84^. Taxonomic assignment was conducted using the Silva database v128^85^ and the RDP Classifier^86^ with a bootstrap confidence threshold of 70%.

Samples with fewer than 999 reads in total were excluded. Chloroplast and mitochondrial sequences, OTUs not present in more than one sample, and OTUs with an abundance of less than 0.02% were pruned. Following these filtering steps, all 45 samples were retained for statistical analysis. The final dataset included 971,644 reads (average number of reads: 21,592; range: 13,663– 28,638) corresponding to 177 OTUs.

R (version 4.0.2) and the R-package “phyloseq” (version 1.32.0^87^,) were used for data visualization and analyses. Selected alpha diversity indices (Observed, Chao1 and Shannon) were calculated with the R-package “phyloseq”. Means of bacterial richness (Observed, Chao1) and diversity (Shannon) estimates were compared with the aim of evaluating the influence of the factor Exercise in both genotypes (Table 1). Data were checked for normality by analyzing the model residuals with the Shapiro-Wilk normality test before pairwise comparisons were conducted, all implemented in the package “rstatix” (version 0.6.0^88^). Statements of statistical significance were based upon *p*-values < 0.05. The adonis function of the “vegan” package (version 2.5.6^89^) was used for permutational multivariate analysis of variance (PERMANOVA) on Bray–Curtis distances to quantify the contribution of the factors Genotype and Exercise to the differences in microbial composition of the samples. A Bray–Curtis dissimilarity-based principal coordinate analysis (PCoA) was performed to visualize differences in the fecal microbiota composition of mice. Differentially abundant OTUs between both treatment groups at different time points were identified with the help of the R package “DESeq2” (version 1.28.1), which uses tests based on the negative binomial distribution^90^. Raw *p*-values were adjusted using the method of Benjamini and Hochberg^91^ to control a false discovery rate (FDR) of 5%. Additionally, a cutoff for the log_2_-fold change of ±1 was set.

### Statistics

GraphPad Prism Version 9 was used for statistics. A repeated measure or regular 2-way ANOVA was employed for behavioral and histological parameters to compare datasets containing more than two groups followed by Sidak’s post-hoc test. Statistical significance was ascribed to p-values less than 0.05, denoted with an asterisk (*). A *p*-value between 0.05 and 0.1 indicated a trend. Scientists performing behavioral or histological ratings were blinded to the genotype and treatment.

## Supplementary information

Supplementary information

## Data Availability

The data that support the findings of this study are available from the corresponding author upon reasonable request.
