# Supplementary material for: Depending on intensity, exercise improved or worsened pathology in a model of prodromal Parkinson’s disease
Source: NPJ Parkinsons Dis. 2025 Nov 23;11:335. doi: 10.1038/s41531-025-01200-y (PMC12644703; doi:10.1038/s41531-025-01200-y)
Supplement: Supplementary file 1 — Supplementary information [file 41531_2025_1200_MOESM1_ESM.pdf]

Supplementary information

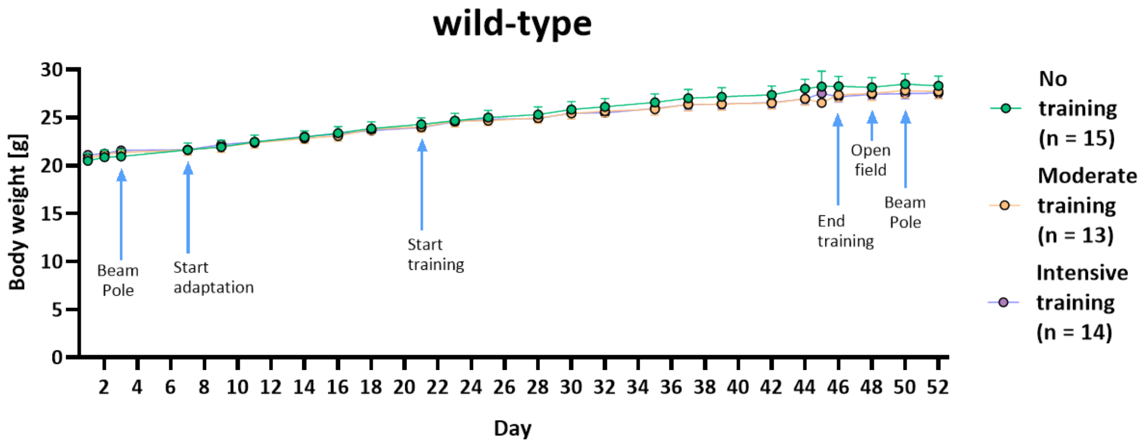

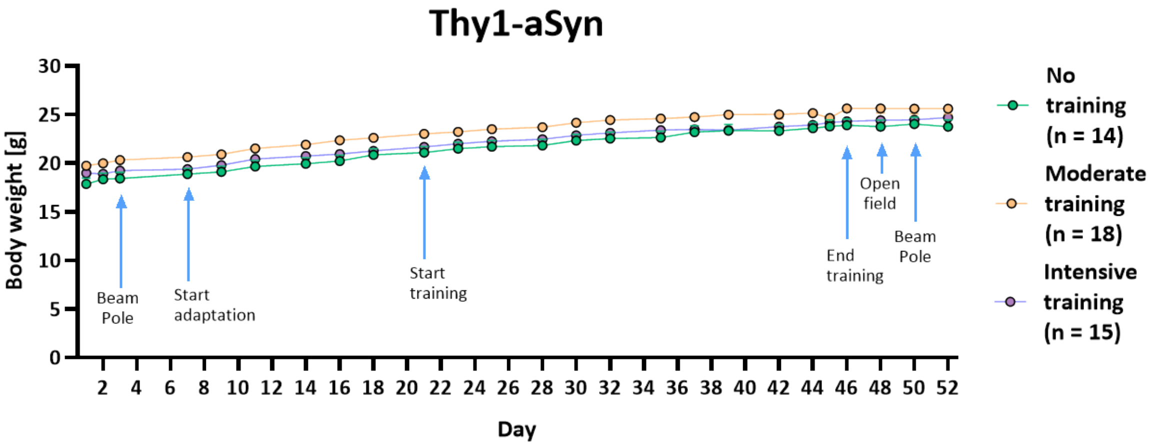

Figure S1. Body weight development in wild-type (WT) control and transgenic (TG) mice are depicted. Raw weight data is available upon request.

Illustration of phosphorylated human alpha-synuclein (paSyn) in the basolateral amygdala and hippocampal CA1 region of Thy1-aSyn mice for anatomical context only and not for between-group intensity comparison.

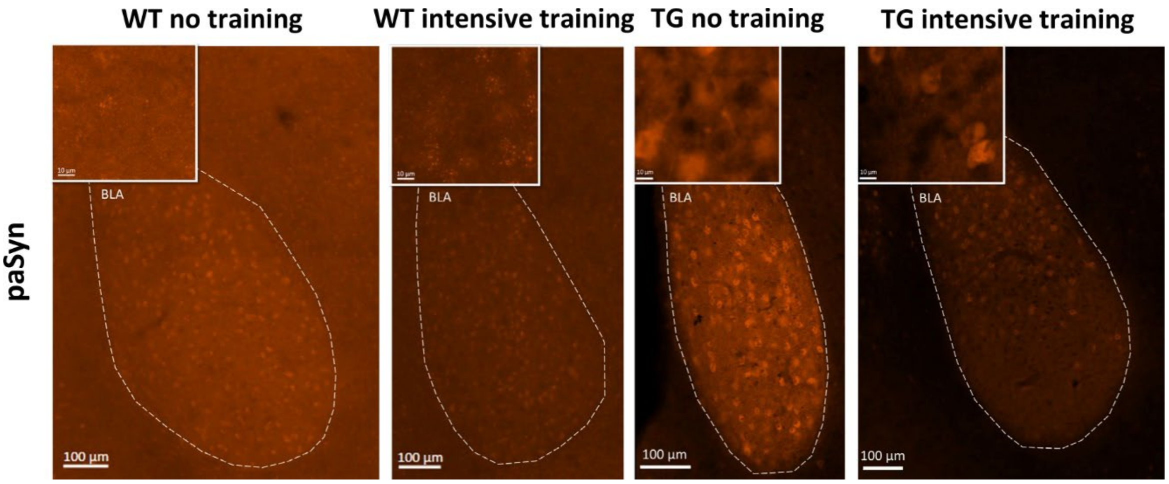

Figure S2. Immunofluorescent staining of Ser129 phosphorylated human alpha-synuclein (paSyn) in the basolateral amygdala (BLA) of wild-type (WT) control and transgenic (TG) mice that received no or intensive training. TG images are identical as in Fig. 5a, whereas WT images were rescaled to visualize tissue outlines; 200x total magnification, scale bars are as indicated (100 µm, insets: 10 µm); display range: 0-400 digital gray value in WT images (20-fold reduction compared to TG), 0-8000 digital gray value in TG images.

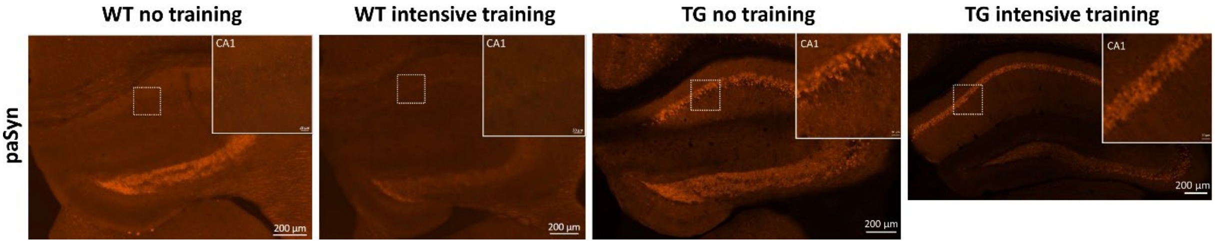

Figure S3. Immunofluorescent staining of Ser129 phosphorylated human alpha-synuclein (paSyn) in the hippocampal CA1 region of wild-type (WT) control and transgenic (TG) mice that received no or intensive training. TG images are identical as in Fig. 6a, whereas WT images were rescaled to visualize tissue outlines; 200x total magnification, scale bars are as indicated (200 µm, insets: 20 µm); display

range: 0-500 digital gray value in WT images (10-fold reduction compared to TG), 0-5000 digital gray value in TG images.
